# Supplementary material for: Multi-omics analysis of miRNA-mediated intestinal microflora changes in crucian carp Carassius auratus infected with Rahnella aquatilis
Source: Front Immunol. 2024 Feb 15;15:1335602. doi: 10.3389/fimmu.2024.1335602 (PMC10902443; doi:10.3389/fimmu.2024.1335602)
Supplement: Supplementary file 1 [file Table_1.docx]

**Supplemented Table 1**

Selected miR-205 sequences for 26 different species from the miRBase database

| Category | Abbreviation | Species | MiRRase ID |
| --- | --- | --- | --- |
| Mammalia | hsa-mir-205 | *Homo sapiens* | MI0000285 |
|  | mmu-mir-205 | *Mus musculus* | MI0000248 |
| Salmoniformes | ssa-mir-205a-1 | *Salmo salar* | MI0026594 |
| Beloniformes | ola-mir-205 | *Oryzias latipes* | MI0019453 |
| Cypriniformes | ipu-mir-205 | *Ictalurus punctatus* | MI0024563 |
|  | dre-mir-205 | *Danio rerio* | MI0001378 |
|  | cau-mir-205 | *Carassius auratus* | **In this study** |
|  | ccr-mir-205 | *Cyprinus carpio* | MI0023365 |
| Amphibian | xtr-mir-205a | *Xenopus tropicalis* | MI0004950 |
| Perciformes | abu-mir-205-1 | *Astatotilapia burtoni* | MI0033757 |
|  | mze-mir-205-1 | *Metriaclima zebra* | MI0034053 |
|  | oni-mir-205-1 | *Oreochromis niloticus* | MI0034567 |
|  | nbr-mir-205-1 | *Neolamprologus brichardi* | MI0034313 |
|  | pny-mir-205-1 | *Pundamilia nyererei* | MI0034829 |
| Aves | tgu-mir-205 | *Taeniopygia guttata* | MI0013846 |
|  | cli-mir-205a | *Columba livia* | MI0030011 |
|  | gga-mir-205a | *Gallus gallu* | MI0001267 |
| Reptilia | aca-mir-205a | *Anolis carolinensis* | MI0018808 |
|  | ami-mir-205a | *Alligator mississippiensis* | MI0029791 |
|  | pbv-mir-205a | *Python bivittatus* | MI0030281 |
|  | cpi-mir-205a | *Chrysemys picta* | MI0029531 |
|  | oha-mir-205a | *Ophiophagus hannah* | MI0031406 |
| Petromyzontiformes | pma-mir-205a | *Petromyzon marinus* | MI0017107 |
| Tetraodontiformes | tni-mir-205 | *Tetraodon nigroviridis* | MI0003282 |
|  | fru-mir-205 | *Takifugu rubripes* | MI0003281 |
| Gadiformes | gmo-mir-205-1 | *Gadus morhua* | MI0036060 |
