# Supplementary material for: Multi-omics analysis of miRNA-mediated intestinal microflora changes in crucian carp Carassius auratus infected with Rahnella aquatilis
Source: Front Immunol. 2024 Feb 15;15:1335602. doi: 10.3389/fimmu.2024.1335602 (PMC10902443; doi:10.3389/fimmu.2024.1335602)
Supplement: Supplementary file 2 [file Table_2.docx]

**Supplemented Table 2**

Primer information of the Intestinal immune-related genes in *C. auratus*

| Gene bank accession | Primer name | Primer sequence | Product size(bp) | Annealing range(℃) |
| --- | --- | --- | --- | --- |
|  |  |  |  |  |
| JX507739.1 | *MHC Ⅱ-F* | GCAGACCTAATGCTCAGTTCCT | 132 | 58 |
|  | *MHC Ⅱ-R* | AGAACTCATATGCGCTGCAC |  |  |
| XM_026212199.1 | *C3-F* | CATGTTGGAGATGAAGTGCG | 157 | 58 |
|  | *C3-R* | CAACATCCACATCAGTGCCT |  |  |
| XM_026233480.1 | *TNF-α-F* | CCTGACATGAGTGAGCAGCA | 116 | 55 |
|  | *TNF-α-R* | GTGTCCAGTGTGCCTCTGTT |  |  |
| XM_ 026238024.1 | *LysC-F* | GAGATGAGGGTGGCTGTTGT | 101 | 55 |
|  | *LysC-R* | TCTCGCTTGAAGATACGGGC |  |  |
| AB894120 | *NF-κB-F* | TTGCAGCCAAAGATCGAAATGA | 146 | 58 |
|  | *NF-κB-R* | TCGAAAGCAGGTCTTGGCATA |  |  |
| XM_026269571.1 | *TLR3-F* | GACGCCAGCTACAACTCTTTA | 186 | 58 |
|  | *TLR3-R* | GAACGGCTCTCCATTTAGCTT |  |  |
| XM_026196237.1 | *TGF-β-F* | CTGGGCTGGAAGTGGATAC | 188 | 58 |
|  | *TGF-β-R* | TAAACGATGGGCAGTGGGT |  |  |
| XM_026249409 | *IL-15-F* | ACTTCCTCGCCTTCATCGT | 174 | 58 |
|  | *IL-15-R* | CGGGCAATAGAGCACTTAGAC |  |  |
| XM_026267672.1 | *F2-F* | CCCAGAACATCAGCAAACATCA | 149 | 55 |
|  | *F2-R* | TGGGGGAAGTTGCTTTTCCA |  |  |
| NM_001007208.2 | *F5-F* | AACAGGACGATGCCGTACAA | 103 | 58 |
|  | *F5-R* | GTCCACGTACGACAGTCAGA |  |  |
| MK246404 | *MyD88-F* | ACAGTCGCCGAAATGATGGA | 112 | 58 |
|  | *MyD88-R* | CTGTTGCCTCTGGACGAGTT |  |  |
