# Supplementary material for: Multi-omics analysis of miRNA-mediated intestinal microflora changes in crucian carp Carassius auratus infected with Rahnella aquatilis
Source: Front Immunol. 2024 Feb 15;15:1335602. doi: 10.3389/fimmu.2024.1335602 (PMC10902443; doi:10.3389/fimmu.2024.1335602)
Supplement: Supplementary file 3 [file Table_3.docx]

**Supplemented Table 3**

High-throughput sequencing data statistics of intestinal in *C. auratus* infected with *R. aquatilis*.

| Groups | Samples | Raw reads | Clean reads | Q30(%) |
| --- | --- | --- | --- | --- |
| Control | IC1 | 20,225,850 | 19,567,006 | 95.96 |
|  | IC2 | 13,949,311 | 13,580,107 | 96.57 |
|  | IC3 | 14,841,942 | 14,174,605 | 96.99 |
| Infection | IT1 | 12,981,132 | 12,149,705 | 96.29 |
|  | IT2 | 14,461,606 | 13,774,711 | 96.66 |
|  | IT3 | 14,243,630 | 13,594,730 | 96.64 |
| Total |  | 90703471 | 86840864 |  |
