# Supplementary material for: Multi-omics analysis of miRNA-mediated intestinal microflora changes in crucian carp Carassius auratus infected with Rahnella aquatilis
Source: Front Immunol. 2024 Feb 15;15:1335602. doi: 10.3389/fimmu.2024.1335602 (PMC10902443; doi:10.3389/fimmu.2024.1335602)
Supplement: Supplementary file 4 [file Table_4.docx]

**Supplemented Table 4**

Differential expression of miRNAs in *C. auratus* intestinal infected with *R. aquatilis*.

| miRNA name | P-value | Fold Change | Up/Down  Regulation |
| --- | --- | --- | --- |
| miR-10b-3p | 0.006 | -1.09 | down |
| miR-129 | 0.028 | 0.31 | down |
| miR-133-3p | 0.001 | 1.92 | up |
| miR-133b-3p | 0.001 | 1.90 | up |
| miR-203 | 0.01 | -1.94 | down |
| miR-203a | 0.01 | -1.91 | down |
| miR-203b-3p | 0.0004 | -2.72 | down |
| miR-205 | 0.04 | -1.61 | down |
| miR-205b-5p | 0.04 | -1.61 | down |
| miR-206 | 1.49E-08 | 7.16 | up |
| miR-206-3p | 2.28E-08 | 7.17 | up |
| miR-499 | 0.0003 | 1.94 | up |
| miR-499-5p | 0.0003 | 1.94 | up |
| miR-499b-5p | 0.0005 | 1.98 | up |
| novel_miR_109 | 0.04 | -3.97 | down |
| novel_miR_129 | 0.03 | -4.53 | down |
| novel_miR_234 | 0.004 | 1.78 | up |
| novel_miR_485 | 0.002 | 1.75 | up |
| novel_miR_982 | 0.007 | -2.28 | down |
| novel_miR_1276 | 5.10E-07 | 5.15 | up |
| novel_miR_1631 | 8.68E-09 | 7.72 | up |
| novel_miR_1652 | 5.08E-07 | 5.15 | up |
