# Supplementary material for: Multi-omics analysis of miRNA-mediated intestinal microflora changes in crucian carp Carassius auratus infected with Rahnella aquatilis
Source: Front Immunol. 2024 Feb 15;15:1335602. doi: 10.3389/fimmu.2024.1335602 (PMC10902443; doi:10.3389/fimmu.2024.1335602)
Supplement: Supplementary file 5 [file DataSheet_1.docx]

**
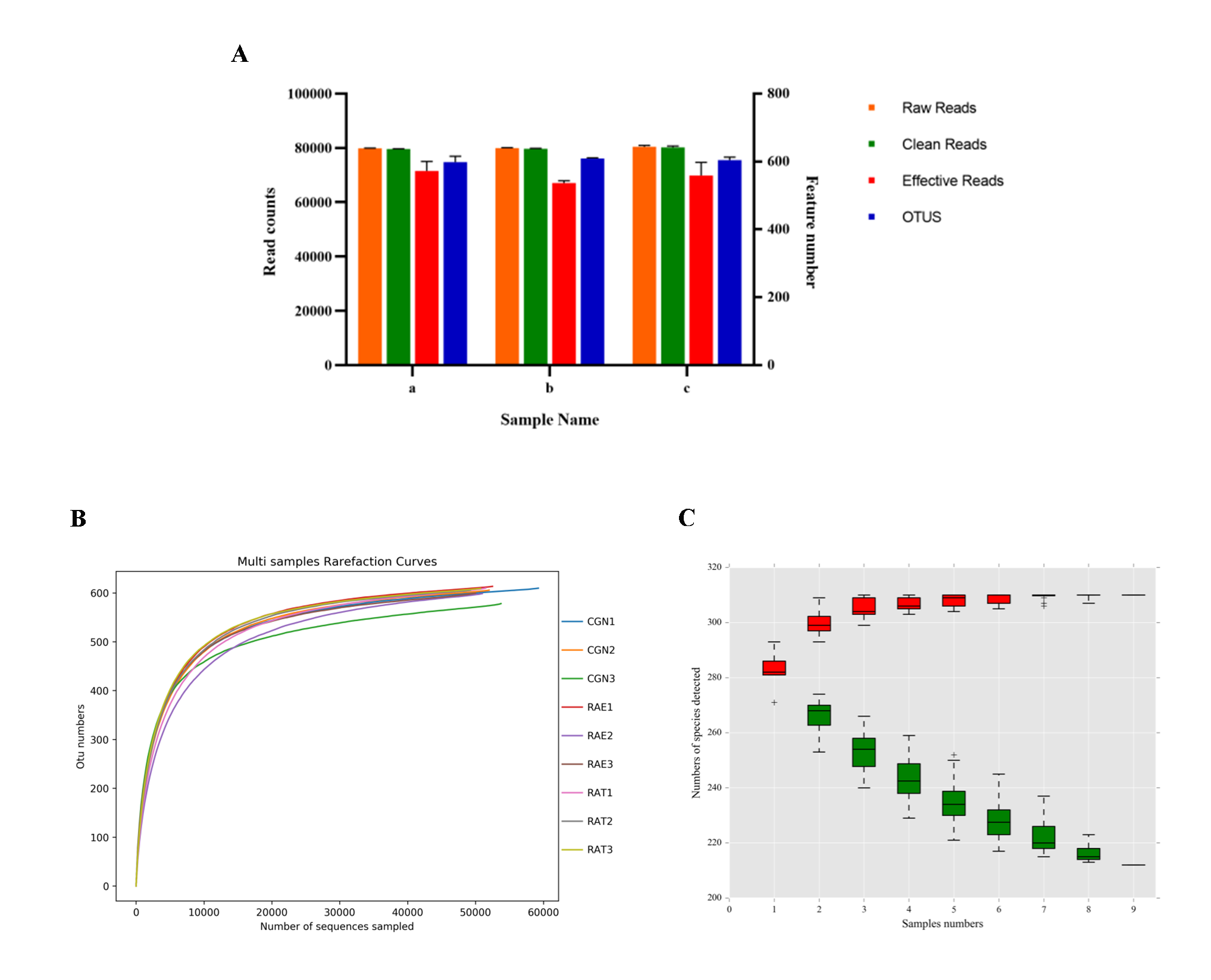
**

**Supplemented Fig. 1.** Overview of 16s rRNA sequencing in the intestine of *C. auratus*. (A) Statistics of the sequence information of the intestinal content. (B) Dilution curve of the intestinal content. (C) Species accumulation curve of intestinal content samples.
