# Supplementary material for: Multi-omics analysis of miRNA-mediated intestinal microflora changes in crucian carp Carassius auratus infected with Rahnella aquatilis
Source: Front Immunol. 2024 Feb 15;15:1335602. doi: 10.3389/fimmu.2024.1335602 (PMC10902443; doi:10.3389/fimmu.2024.1335602)
Supplement: Supplementary file 6 [file DataSheet_2.docx]

**
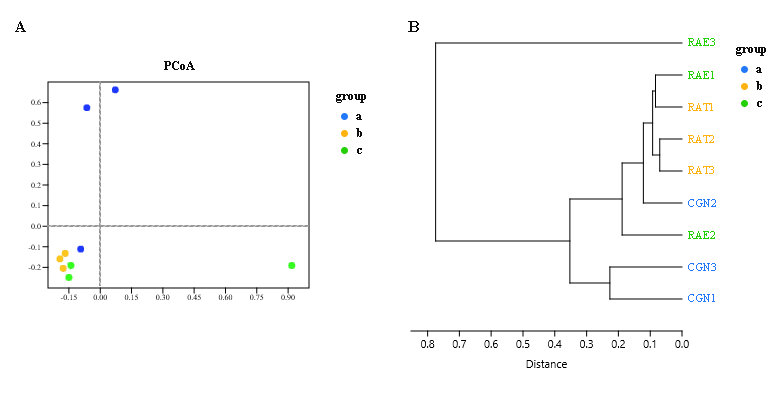
**

**Supplemented Fig. 2**. Analysis of intestinal microflora Bate diversity of *C. auratus* infected with *R. aquatilis*. (A) PCoA analysis of the intestinal microflora. (B) UPGMA cluster tree analysis of intestinal microflora.
