# Supplementary material for: Multi-omics analysis of miRNA-mediated intestinal microflora changes in crucian carp Carassius auratus infected with Rahnella aquatilis
Source: Front Immunol. 2024 Feb 15;15:1335602. doi: 10.3389/fimmu.2024.1335602 (PMC10902443; doi:10.3389/fimmu.2024.1335602)
Supplement: Supplementary file 7 [file DataSheet_3.docx]

**
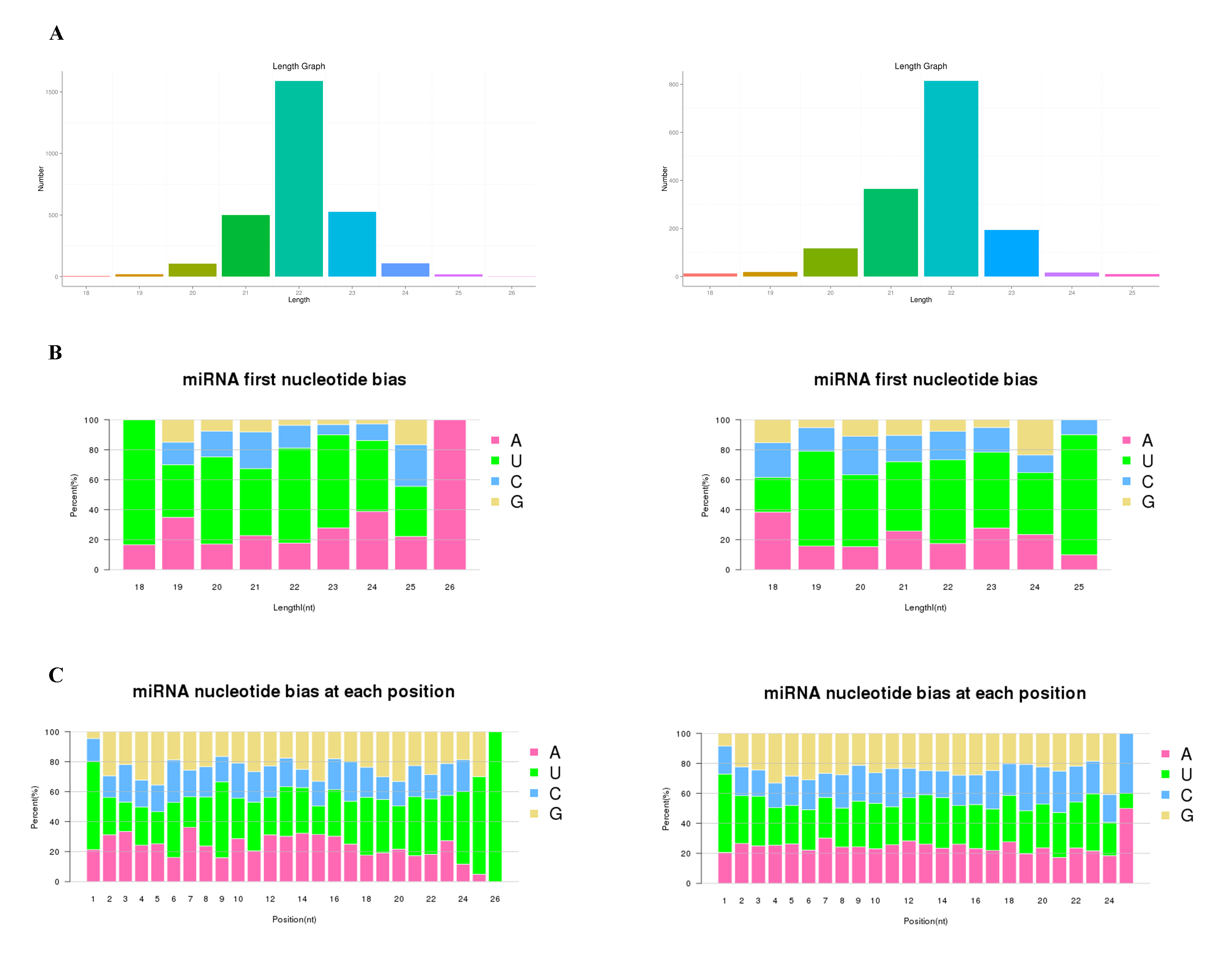
**

**Supplemented Fig. 3**. Distribution of known miRNAs (Left) and novel miRNAs (Right) in *C. auratus* intestinal infected with *R. aquatilis*. (A) Length distribution of miRNAs. (B) First base distribution of miRNAs. (C) Nucleotide bias at each position of miRNAs.
